# Challenges in funding and developing genomic software: roots and remedies

**DOI:** 10.1186/s13059-019-1763-7

**Published:** 2019-07-29

**Authors:** Adam Siepel

**Affiliations:** Simons Center for Quantitative Biology, Cold Spring Harbor Laboratory, Cold Spring Harbor, NY 11724 USA

## Abstract

The computer software used for genomic analysis has become a crucial component of the infrastructure for life sciences. However, genomic software is still typically developed in an ad hoc manner, with inadequate funding, and by academic researchers not trained in software development, at substantial costs to the research community. I examine the roots of the incongruity between the importance of and the degree of investment in genomic software, and I suggest several potential remedies for current problems. As genomics continues to grow, new strategies for funding and developing the software that powers the field will become increasingly essential.

A traveler in late-eighteenth-century England who passed through the town of Slough—located just west of London and not far from present-day Heathrow Airport—might have come upon a massive 40-ft-long telescope, suspended in a wooden frame more than 50 ft tall (Fig. 1a). The telescope was located at the home of William Herschel and his sister Caroline, two of the greatest astronomers of their day. It was the largest telescope in the world until it was dismantled in 1839. Weighing over 1000 lbs., the “40-ft telescope”, as it was known, was sufficiently impressive to the general public to emerge as a regional tourist attraction. Its audacious scale inspired prominent thinkers and writers of the time, including Erasmus Darwin and William Blake [1, 5].

**Fig. 1:**
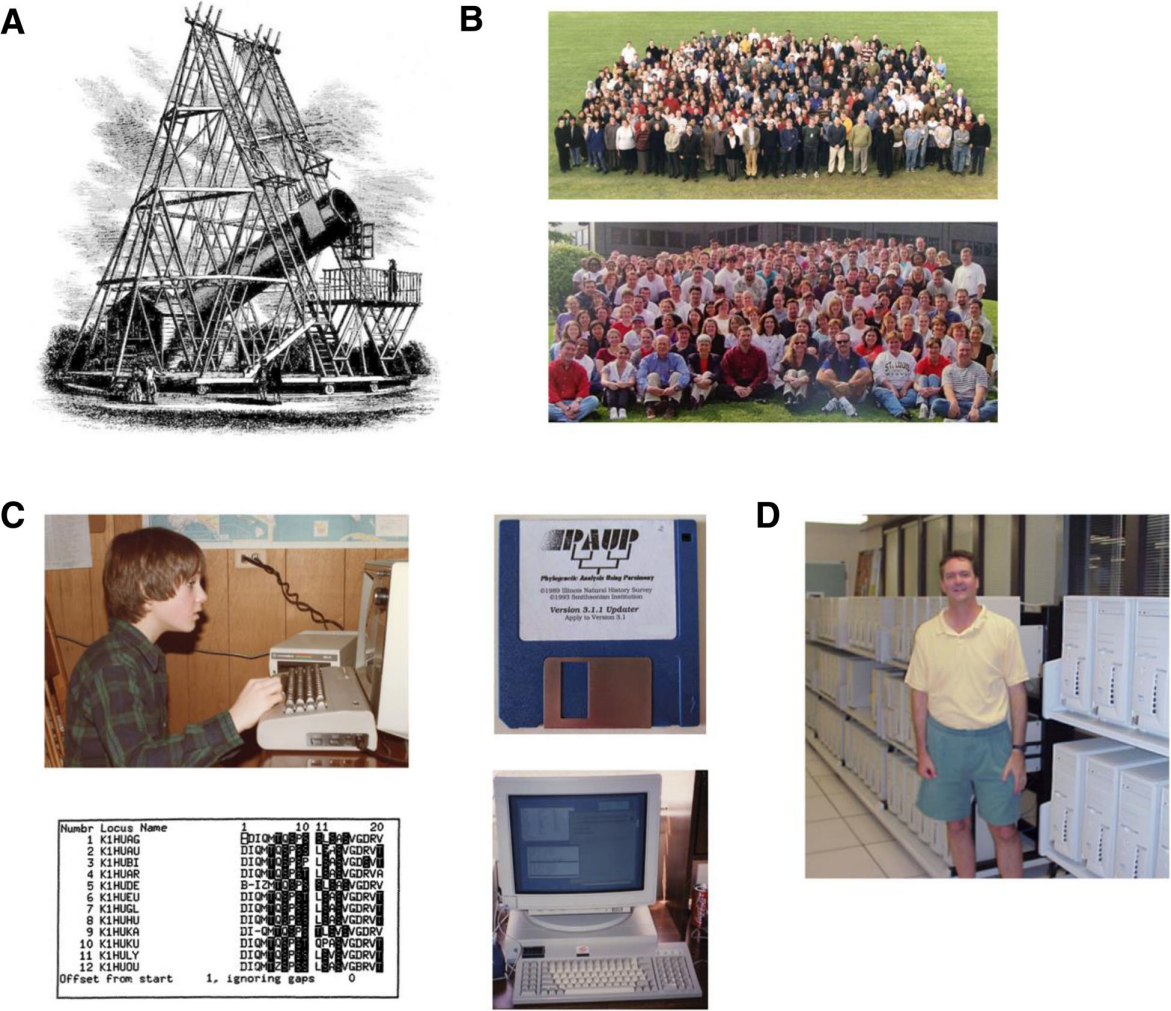
**a** William and Caroline Herschel’s 40-foot telescope in Slough, England, 1789 [1]. **b** Two of the teams of scientists that contributed to the Human Genome Project: (*top*) Sanger Center, Hinxton, UK [2]; (*bottom*) Washington University Genome Sequencing Center, St. Louis, USA, both circa 2000. **c** Some relics of the pre-Internet world: (*clockwise* from *top left*) the author learning to program in BASIC on a Commodore 64 in a cold upstate New York basement, 1983 (*credit*: Virginia Siepel), as one of the millions of children who were introduced to home computers during the 1980s and 1990s, some of whom would go on to write much of the software that powers genomics today; floppy disk for PAUP version 3.1.1,©1993; Sun Microsystems SPARCstation 1 with Mosaic web browser faintly visible on screen, 1994 [3]; screen shot from the MASE alignment program [4]. **d** Prof. David Haussler of UC Santa Cruz with the original Dell computer cluster that his team used to assemble the human genome, 2000. Photo (c) UC Santa Cruz, used with permission

The 40-ft telescope took 5 years to build and was paid for by a grant of £4000 from King George III, who was strongly committed to scientific research throughout his reign. This grant represented a substantial sum at the time, roughly equivalent to £600,000 (about US$800,000) in 2019 [6]. There were no formal mechanisms at the time for grant applications for scientific research. Instead, William Herschel simply approached the King directly with a request for royal patronage. The 40-ft telescope is one of the earliest examples of government investment in the infrastructure for scientific research, to enable a project that simply would not have been possible with private funds alone.

The model of government investment in scientific infrastructure became increasingly well-established throughout the 19th and 20th centuries, culminating in the “Big Science” of the World War II and Post-War eras. Science in modern times has been dominated, in many ways, by these massive public investments. Prominent examples include the Manhattan project (equivalent to $22 billion in 2016 [7, 8]), the Apollo program (equivalent to $107 billion [9]), the Space Shuttle program (equivalent to $219 billion [10]), the Large Hadron Collider (equivalent $4.8 billion [11]) and, more pertinent to this article, the Human Genome Project (equivalent to $5.0 billion [12]; Fig. 1b).

Indeed, we now live in a world where much of the day-to-day work in science depends on a publicly funded infrastructure. In particular, many working in genomics rely heavily on data sets such as those generated by the ENCODE, Roadmap Epigenomics, 1000 Genomes, Genotype-Tissue Expression (GTEx), The Cancer Genome Atlas (TCGA), Genetic European Variation in Disease (GEUVADIS), and most recently, Human Cell Atlas projects. We store and search sequence data using GenBank, EMBL-Bank, DDBJ, UniProt, and Pfam, examine three-dimensional protein structures in PDB or EMDB, scour the literature using PubMed, and view genomic annotations using the UCSC Genome Browser and Ensembl Browser. All these resources have been maintained for decades either directly by government agencies or through long-term public funding to universities and research institutes.

The computer software on which millions of scientists rely for genomic analysis is no less an essential part of the infrastructure of biological research than large shared data sets or public databases, yet the model for funding and developing computer software differs substantially. Most widely used genomic software is developed by independent investigators working in academic or not-for-profit institutions with support from government grants. This software is generally freely available to the community, typically with no subscription or licensing fees and nonrestrictive terms of use. At the same time, it is often meagerly funded, unreliable, hard-to-use, poorly documented, and/or poorly supported. How did we, as a community, arrive at this odd situation? Why is scientific software supported differently from other forms of scientific infrastructure? Why are adequate funds not set aside for this important work?

In this article, I offer my perspective on the unique problem of funding and developing software for genomics, based on my 25 years in the field—as a developer and user of software, a professional programmer and principal investigator, an applicant for and reviewer of grant proposals, and an employee of government, university, and private research institutions. I first examine what makes genomic software development unusual and how the field has come to be the way it is. Overall, I argue that, despite some important strengths of our current model for software development, we as a community have “painted ourselves into a corner” in terms of developing robust, well-engineered software and are paying for it; we are, in a sense, addicted to free software. Finally, I suggest some possible remedies that attempt to strike a balance between addressing important deficiencies in the current model and maintaining its core strengths. My discussion of these topics necessarily have a US bias, but I believe that many of my points are internationally valid. Also, although this article focuses on genomics, similar trends occur in other areas of computational biology, such as structural biology and proteomics, as well as in some other areas of scientific software development.

## Software for genomics is critical to the research infrastructure for the life sciences

During the past 25 years, genomic software development has grown from an obscure cottage industry to an essential part of the infrastructure of biological research. Researchers across the globe rely on computational tools for read mapping, genome assembly, multiple alignment, phylogenetics, population genomics, and visualization of genomic data, among many other applications. Importantly, these tools are no longer used only by genomic specialists, but across all the life sciences, including disparate fields such as ecology and evolution, molecular and cell biology, clinical genetics, plant breeding, biophysics, and bioengineering. To take one measure of impact, the papers describing popular genomic software tools are among the most highly cited publications in the scientific literature [13, 14]. For example, Table 1 lists 66 well-known genomic software tools, from various application areas, each of which has been cited at least 2000 times and, in some cases, many tens of thousands of times (for reference, only about 1 in 100,000 scientific papers is cited more than 2000 times^,^ based on estimates from Open Academic Graph [152], a bibliographic database of ~ 700 million publications (analysis restricted to biology-related publications)). Indeed, nowadays it is rare to encounter a scientific publication that makes use of DNA, RNA, or protein sequences but does not reference one or more tools of this type.

**Table 1:** Highly cited genomic software tools

Program name	Year^a^	Primary institution(s)^b^	Primary funding source(s)^c^	Refs.^d^	Citations^e^
Homology searching and alignment					
FASTA	1988	U. Virginia, NIH	*NA*	[15]	13,496
CLUSTAL	1988	Trinity College, Dublin; EMBL, Heidelberg; EBI	European Community Biotechnology Action Programme	[16–20]	94,789
BLAST	1990	NCBI	NIH	[21]	75,328
PSI-BLAST	1997	NCBI	NIH	[22]	69,604
HMMer	1998	Washington U., St. Louis	NIH, HHMI	[23–25]	8,836
T-Coffee	2000	Nat. Inst. Med. Res., London	Swiss Nat’l Science Fnd.	[26]	6,247
BLAT	2002	UC Santa Cruz	NIH, HHMI	[27]	6,911
MUSCLE	2004	drive5.com	*NA*	[28]	24,261
MAFFT	2013	Kyoto U.	Ministry of Education, Culture, Sports, and Technology of Japan	[29–33]	21,486
Phylogenetic modeling and tree inference					
PHYLIP	1980	U. Washington	NIH, NSF	[34]	21,851
MacClade	1986	Sinauer Assoc.	(Commercial)	[35]	10,255
PAUP	1989	Illinois Nat. Hist. Survey, Sinauer Assoc.	(Commercial)	[4]	62,807
PAML	1993	UC Berkeley, Univ. College London	NSF of China, NIH, NSF	[36, 37]	11,375
MEGA	1993	Penn. State U., Arizona State U.	NIH, NSF, Burroughs-Wellcome	[38–42]	119,268
Mr. Bayes	2001	U. Rochester, Uppsala U.	NSF	[43–45]	52,742
Mesquite	2001	U. Arizona, U. British Columbia	Packard, NSF	[46]	7,693
PhyML	2003	CNRS, Montpellier	Montpellier Genopole, InterEPST Bioinformatics Program	[47–50]	24,614
PHAST	2004	UC Santa Cruz, Cornell	NSF, Packard, NIH	[51–55]	4,690
RAxML	2004	Technical U. Munich	Heidelberg Institute for Theoretical Studies	[56–59]	27,550
HyPhy	2005	UC San Diego, NC State	*NA*	[60]	2,159
BEAST	2007	U. Auckland, U. Edinburgh	Wellcome Trust, Royal Society	[61, 62]	12,027
FastTree/FastTree2	2009	Lawrence Berkeley Nat’l Lab, UC Berkeley	DOE, GTL Program	[63, 64]	5,308
Gene prediction, motif finding, and RNA folding					
MEME	1994	UC San Diego	NIH, NSF	[65–69]	11,790
Genscan	1997	Stanford	NIH, NSF	[70]	4,061
tRNAscan-SE	1997	Washington U., St. Louis	*NA*	[71]	7,559
Vienna package	2003	Institute for Theoretical Chemistry, Austria	Austrian Science Fund	[72–74]	4,781
Visualization					
Jalview	1996	EBI, Sanger, Oxford	BBSRC	[75, 76]	5,895
TreeView	1998	Stanford	NIH	[77]	17,796
UCSC Genome Browser	2000	UC Santa Cruz	NIH, DOE, HHMI	[78–82]	11,365
ENSEMBL Browser	2000	EBI, Sanger	Wellcome Trust, NIH, EMBL	[83–87]	5,235
Cytoscape	2003	Inst. Systems Biology, Whitehead Inst., UC San Diego	Pfizer, NIH, NSF	[88–90]	17,862
IGV	2011	Broad	NIH	[91]	4,678
Statistical and population genomics					
STRUCTURE	2000	Oxford	NIH, Burroughs-Wellcome, BBRC	[92, 93]	30,948
PHASE/fastPHASE	2001	Oxford, U. Washington	Wellcome Trust, BBSRC, Engineering and Physical Sciences Research Council	[94–96]	10,073
ms	2002	U. Chicago	*NA*	[97]	2,119
PolyPhen	2002	EMBL, Max Delbrück Center for Mol. Med., Engelhardt Inst. Mol. Biol.	NIH	[98–100]	11,136
SIFT	2003	Fred Hutchinson Cancer Res. Ctr.	NIH	[101, 102]	7,024
EIGENSTRAT	2006	Harvard, Broad	Millenium Pharmaceuticals, Burroughs Wellcome	[103]	6,812
PLINK	2007	MGH, Broad, U. Hong Kong	NIH	[104, 105]	17,938
TASSEL	2007	USDA-ARS, Cornell	USDA-ARS, NSF	[106]	2,609
BEAGLE	2007	U. Auckland	University of Auckland Research Committee, NIH	[107, 108]	2,997
IMPUTE/IMPUTE2	2007	Oxford	Wellcome Trust, NIH	[109, 110]	4,930
VCFtools	2011	Sanger	Medical Research Council, British Heart Foundation, Wellcome Trust, NIH	[111]	3,133
CADD	2014	U. Washington	NIH	[112]	2,353
Functional genomics, annotations, and transcriptomics					
Gene Ontology	2000	UC Berkeley, Stanford	NIH, Astra Zeneca	[113]	22,898
GSEA	2005	Broad	*NA*	[114]	16,135
MACS/MACS2	2008	Dana-Farber, Harvard	NIH	[115, 116]	5,965
TopHat/Cufflinks	2009	U. Maryland	NIH, NSF	[117–120]	28,242
ChromHMM	2010	MIT, Broad	NSF, NIH	[121–123]	3,977
BEDtools	2010	U. Virginia	NIH, Burroughs-Wellcome	[124, 125]	7,137
edgeR	2010	Garavan Inst. Med. Res., Walter & Eliza Hall Inst. Med. Res., Australia	NHMRC	[126]	9,992
Trinity	2011	MIT, Broad	NIH, US-Israel Binational Science Foundation	[127]	7,178
DEseq/DEseq2	2012	EMBL	*NA*	[128, 129]	16,355
Assembly, read mapping, and base/variant calling					
Staden package	1977	LMB	*NA*	[130–134]	5,029
Phred	1993	Washington U., St. Louis, U. Washington	NIH	[135, 136]	12,172
MAQ	2008	Sanger	Wellcome Trust	[137]	2,777
ALLPATHS/ALLPATHS-LG	2008	Broad, MGH	NIH	[138, 139]	2,079
Velvet	2008	EBI	EMBL	[140]	7,635
Bowtie/Bowtie2	2009	U. Maryland	NIH	[141, 142]	26,607
BWT	2009	Sanger	Wellcome Trust	[143]	17,546
SOAP2	2009	Beijing Genomics Inst., U. Southern Denmark	National Natural Science Foundation of China, Danish Natural Science Research Council	[144]	2,818
SAMtools	2009	Sanger	Wellcome Trust, NIH	[145]	17,811
ABySS	2009	Genome Sciences Centre, Vancouver, BC	Genome Canada, Genome British Columbia, British Columbia Cancer Foundation	[146]	2,761
GATK	2010	Broad, MGH	NIH	[147]	9,291
SOAPdenovo/SOAPdenovo2	2010	Beijing Genomics Inst.	Chinese Academy of Science, National Natural Science Foundation of China	[148, 149]	4,295
STAR	2013	CSHL	NIH	[150, 151]	6,013

^a^ Approximate first year available, or year of first publication if unknown

^b^Institutions most central in supporting project, or affiliations of first and last authors of first publication if unknown. *Broad* Eli & Edythe Broad Institute of MIT & Harvard, USA; *CNRS* Centre National de la Recherche Scientifique, France; *CSHL* Cold Spring Harbor Laboratory, USA; *EBI* European Bioinformatics Institute; *EMBL* European Molecular Biology Laboratory; *HSPH* Harvard School of Public Health, USA; *LMB* Laboratory of Molecular Biology, UK; *MGH* Massachusetts General Hospital, USA; *Sanger* Wellcome Trust Sanger Institute, UK

^c^ *BBSRC* Biotechnology & Biological Sciences Research Council, UK; *HHMI* Howard Hughes Medical Institute, US; *NA* not applicable; *NCBI* National Center for Biotechnology Information, US; *NHMRC* The National Health & Medical Research Council, Australia; *NIH* National Institutes of Health, US; *NSF* National Science Foundation, US; *USDA-ARS* United States Department of Agriculture - Agriculture Research Service; *Packard* David & Lucile Packard Foundation

^d^ Most highly cited associated publications (at most five)

^e^ Total number of citations, obtained from Google Scholar on Feb. 22, 2019

Because the reach of genomics software is so vast, it is difficult to measure its economic importance. Nevertheless, the US government spends at least ~ $16 billion per year on basic research in the life sciences (spending on research and development by the US Federal government was estimated at $118 billion in 2017, of which $32 billion was dedicated to basic research. The life sciences account for approximately half of all spending, suggesting approximately $16 billion is spent on basic life sciences research [153]). If even 10% of these funds are devoted to projects that rely in part on genomics and genomic software, which seems plausible, then this software would be instrumental in supporting more than a billion dollars per year in research. Furthermore, total R&D expenditures in the US are estimated at about four times those of the federal government, and scaling up to worldwide R&D expenditures requires about another factor of three. (Total R&D expenditures in the US, including those in the private sector and at other governmental levels, are estimated at about $500 billion annually. The US leads the world in spending on science, but China is not far behind, and several other countries—including Japan, Germany, South Korea, France, and the UK—also account for substantial amounts. Together, the top ten countries spend about $1.5 trillion per year on R&D [154]). Therefore, a rough calculation suggests that the worldwide research that depends, at least in part, on genomic software is likely to cost tens of billions of dollars annually.

## Software for genomics lacks a sustainable model for development and maintenance

Despite the overwhelming importance of genomic software, there is broad agreement among practitioners that the current model for its development has serious flaws. As noted above, most genomic software is developed by academic groups and funded by government grants, yet there are relatively few dedicated granting opportunities for genomic software development, and those that exist have relatively low levels of funding (see Table 2 for examples of recent and current funding programs). More typically, software development efforts in genomics have to be cloaked as research, for example, by describing the development of a software tool as a single aim or sub-aim of a research grant that is ostensibly focused on biological discovery. Additional funding for computational genomics has been made available through consortium projects, community databases, and browsers (for example, through U24, U41, and U54 opportunities at the US National Institutes of Health (NIH)), but the scope of this work is often quite constrained. Despite that the most widely used tools have been developed by individual laboratories pursuing investigator-initiated work (Table 1), the funding for projects of this kind remains limited.

**Table 2:** Grant opportunities for genomic software development

Title	Source	Country	Last call	Funding rate
Bioinformatics and Computational Biology	Genome Canada	Canada	2017	CAD$12 M
Cyberinfrastructure Initiative	Canada Foundation for Innovation	Canada	2017	~ CAD$10 M
Research Software Program	CANARIE	Canada	Open	CAD$4.5 M
ELIXIR Tools Platform	ELIXIR	(Europe)	Open	*NA*
Call for Challenges and Unlocking of Technological and Scientific Barriers	Institut Français de Bioinformatique (IFB)	France	Open	*NA*
Accelerating Scientific Discovery	Netherlands eScience	Netherlands	2018	~€1 M
Bioinformatics and Biological Resources Fund	BBSRC	UK	2017	Up to £6 M
Transformative Research Technologies	BBSRC, EPSRC, MRC	UK	2017	Up to £3.5 M
Collaborative Computational Tools for the Human Cell Atlas	Chan-Zuckerberg Initiative	USA	2017	$15 M
Continued Development and Maintenance of Software	NIH	USA	2014	*NA*
Cyberinfrastructure for Sustained Scientific Innovation	NSF (spans Directorates)	USA	Open	$46.5 M
Data-Driven Discovery Investigator Competition	Gordon and Betty Moore Foundation	USA	2014	$22.5 M
Extended Development, Hardening & Dissemination of Technologies in Biomedical Computing, Informatics & Big Data Science	NIH	USA	2014	*NA*
Informatics Technology for Cancer Research	NCI/NIH	USA	2018	*NA*
Infrastructure Capacity for Biology	NSF Division of Biological Infrastructure (DBI)	USA	Open	$40 M
Innovation in Cancer Informatics	Fund for Innovation in Cancer Informatics	USA	Open	~ $1 M
Investigator Initiated Research in Computational Genomics and Data Science	NHGRI/NIH	USA	Open	*NA*
BBSRC-NSF/BIO Lead Agency Opportunity in Bioinformatics and Synthetic Biology	NSF Directorate for Biological Sciences (NSF/BIO), BBSRC	USA/UK	2018	*NA*

*BBSRC* Biotechnology and Biological Sciences Research Council, UK; *EPSRC* Engineering and Physical Sciences Research Council, UK; *MRC* Medical Research Council, UK; *NA* not applicable; *NCI* National Cancer Institute, US; *NHGRI* National Human Genome Research Institute, US; *NIH* National Institutes of Health, US; *NSF* National Science Foundation, US

It is particularly difficult for academic researchers to obtain funding to extend, refine, or support software tools that have already proven to be widely useful to the community—for example, to improve performance, usability, robustness, or documentation, or to provide support for bug fixes and user questions. Except in a few special cases (for example, the Continued Development and Maintenance of Software opportunity previously offered by the NIH; Table 2), grant review panels tend to consider projects of this kind to be insufficiently novel to be supported either by dedicated research grants or as components of grants focused on biological discovery. One might expect that this type of engineering-focused work would more naturally be provided by the private sector, as with laboratory equipment or reagents but, despite decades of anticipation, there is still no thriving commercial market for genomics software. It is true that biotech and pharmaceutical companies often have their own in-house software development groups, but there seems to be, at best, weak demand for these products in the larger research community. Moreover, current trends point in the wrong direction, with several relevant grant opportunities having recently been discontinued (Table 2) and little indication of the emergence of a robust commercial market.

In part owing to these financial limitations, it is difficult to recruit and retain professional software developers in academic settings. Perhaps the most severe challenge is that the salary structures and budget models for academic institutions are generally not set up to accommodate six-figure salaries for workers who are not principal investigators or high-level administrators. As a result, software engineers typically accept a substantial salary reduction—of sometimes 50% or more—for the “privilege” of working in scientific research, as opposed to working for an established or start-up high-tech company. (The average salary for an entry-level software engineer in San Francisco, CA is about $110,000 [155].) Furthermore, academic research institutions often do not provide attractive career paths for software developers, offering them, for example, limited options for career advancement, few awards or accolades, and at most small communities of career-matched peers.

Instead, software development is often done by graduate students and postdoctoral researchers who have other priorities and, in many cases, no direct training in the area. Some principal investigators also devote considerable amounts of their own time to software development, but these activities must be balanced against many other responsibilities, including teaching, mentoring, writing scientific papers, and raising funds. Therefore, genomic software development tends to be done on a low budget, with many short-cuts to software engineering best practices.

Software packages developed in this way tend to be sparsely documented, difficult to install and use, restricted to specific platforms, and unreliable. In addition, the support and maintenance of released packages tends to be inconsistent, typically relying on email contact with busy and distracted principal investigators or trainees, and often effectively ending when a key student or postdoctoral researcher changes jobs. All these factors combine to produce a great deal of wasted time and frustration for the users of genomic software. They also contribute to severe challenges in reproducibility in genomic analysis. Indeed, a recent review of nearly 25,000 “omics” software resources published from 2000 to 2017 found that 26% were no longer accessible through URLs published in the corresponding papers [156]. Among accessible tools, 28% could not be installed, and another 21% were deemed “difficult to install.” Together, it appears that, as a field, we are on an unsustainable path for genomic software development. We do not set aside adequate funding for it, we fail to encourage and enforce good engineering practices, we have inadequate structures for recruiting and retaining the workers we need, and we continually pay a high price in reliability, usability, and performance.

## Other aspects of the infrastructure for genomics have alternative funding models

Interestingly, other aspects of the infrastructure for genomics have followed rather different models. DNA sequencing instruments, for example, have for decades been primarily developed and marketed by companies such as Applied Biosystems (now part of Thermo Fisher Scientific), Illumina, Oxford Nanopore Technologies and, until it was recently absorbed by Illumina, Pacific Biosciences of California. The microarray market was (and remains) similarly commercial, at least following an initial experimental phase, with companies such as Affymetrix (also now part of Thermo Fisher Scientific) and Agilent Technologies dominant. Laboratory equipment is provided by companies such as PerkinElmer, Bio-Rad Laboratories, and Becton Dickinson (BD), and computer hardware is provided by Intel, AMD, Apple, Microsoft, Dell, Samsung, Acer, Hewlett-Packard, and many others. These are areas of technology development with substantial “bricks and mortar” needs, including major manufacturing operations, and they address sufficiently large markets with sufficiently high profit margins such that free enterprise is able to meet the needs of scientific research. Despite the general feeling of corporate skepticism among academic scientists, these companies are viewed, by and large, as positive forces for innovation that are complementary to academic science.

By contrast, large, widely used public databases, such as GenBank, EMBL-Bank, and PDB, tend to be directly supported by government agencies or by long-standing government grants. Even smaller database projects located at universities or private research institutes, such as FlyBase, the Saccharomyces Genome Database (SGD), or the Mouse Genome Database (MGD), tend to have substantial, repeatedly renewed government grants. Thus, it seems that there is an implicit understanding in genomics that the management of large public data sets should be centralized and government-supported, while the hardware and instruments used for generating and analyzing data should be provided by the free market. Why is software different from both?

## Roots: dawn of the modern era for computational genomics

When I started working in computational genomics in 1994, as a research assistant at Los Alamos National Laboratory (LANL), the software landscape in the field had a distinctly different feel. Free software was much less plentiful and co-existed symbiotically with widely used commercial products. In the HIV Sequence Database group in which I worked, we had access to purchased copies of MacClade [35], PAUP [4], and the Genetics Computer Group’s (GCG) Wisconsin Package, alongside free software such as MASE [4], BLAST [21], and PHYLIP [34] (Fig. 1c). In addition, “serious” computational scientists at the time generally used expensive proprietary UNIX systems rather than commodity hardware. Linux was still a hobbyist’s operating system and largely invisible in research settings. Similarly, computer clusters were not yet in wide use; instead, universities and research institutes made heavy use of standalone supercomputers for demanding computations. The World Wide Web was in its infancy and had not yet become essential for day-to-day research.

The field would soon change dramatically. In the mid- and late-1990s, the Internet revolutionized software development and, along with it, computational genomics. The rapid growth of the Internet catalyzed the Open Source Software (OSS) and Free Software movements [157], and the widespread adoption of Linux/GNU operating systems. These platforms, in turn, led to a major shift in research computing away from proprietary Unix systems and toward low-cost Linux systems running on commodity hardware. Computer clusters built from inexpensive components rapidly replaced high-end supercomputers (Fig. 1d). At the same time, the Internet made it much easier, cheaper, and faster to ship software: download buttons replaced telephone orders of floppy disks or CDs. This easy and prolific dissemination of code on the Internet fit well with the ethos of scientific research, which tends to favor openness and shared resources and to view profit-making with suspicion. Soon, there was an explosion of free and open-source software for genomics.

In my view, these trends were intensified by a generational shift in the research science community. By the mid-1990s, the ranks of PhD students and young scientists were swelling with a new cohort that had learned to program computers as children, during the PC boom of the 1980s. These young, computer-savvy researchers saw little point in paying for software that they could write themselves. In addition, many found a subversive excitement in producing their own software and releasing the code, free to anyone, on the emerging Internet. In this brave new world, smart kids could go from an idea to a working implementation to worldwide distribution within days, with no need for investors, marketing teams, or salespeople. Young scientists programmed madly in research laboratories and coffee shops, often at odd hours, communicating by email in a new ultra-networked world, while some of their bosses still occupied a musty world of paper journals, written letters, and landline phones. This generational shift occurred across all of science and engineering, but it was perhaps especially pronounced in biology, where the previous generation—except for a few influential pioneers—had been generally slow to embrace computing technologies.

Whatever its cause, this creative and entrepreneurial spirit helped to generate the rich landscape of free, academic software that we now enjoy in genomics. The “artisanal” model of software development in genomics also has had the benefit of enabling rapid development of new methods, a close coupling of software development and research science, and a kind of esprit de corps among bioinformatic tool developers around the world. Nevertheless, some of the same features that have made the field vibrant and productive have contributed to the difficulty of progressing to a more rigorous and professional model of software development. In particular, the surge of development over the past two decades, done in large part by underpaid workers motivated by pure enthusiasm for their craft, has allowed the field to benefit from a great deal of new software without being forced to reckon with its true costs. Institutions have not been forced to pay professional programmers competitive salaries; grant agencies have not been compelled to set aside appropriate funds for a software infrastructure; and the line items for professional software engineering have not made it into budget models. Thus, genomics has become accustomed to, even addicted to, abundant free software. In a sense, in our idealistic, anti-establishment zeal, we free software warriors have locked computational genomics into an unsustainable financial model.

## Remedies: general principles

What, then, can be done to improve the financial and development landscape for genomic software? I address this question by first advancing some general principles, and then putting forward some more specific implementation strategies.

First, a clearer recognition is needed—at all levels, ranging from research institutions to granting agencies to private companies—that software for genomic analysis is a fundamental component of the infrastructure of genomics and requires a substantial commitment of resources. Software development is no less essential to progress of the field, and no less complex and expensive to carry out, than development of new genomic technologies or large-scale databases.

Second, commitments to the development of new software must be accompanied by ongoing commitments to the maintenance, refinement, and support of widely used tools. Because some tools inevitably remain relevant and widely used for longer than others, mechanisms will be needed for determining which previously funded projects do and do not deserve ongoing support.

Third, grant proposal formats and review criteria must be adapted to accommodate fundamental differences between software development projects and genomic research projects. In particular, proposals for software development projects should be evaluated in a way that gives less weight to innovation and more weight to software engineering practices, as well as to distribution, maintenance, support, documentation, and usability.

Fourth, improved career paths are needed for software developers working in academic research settings. Institutions and grant mechanisms must allow for salaries that are competitive with industry, and better opportunities for career advancement and continuing education.

Fifth, academic researchers and funding agencies must remain open to the possibility that some aspects of software development might be better done by private companies and should consider ways to nurture the development of sustainable business models based on genomic software development.

Sixth, it would be a mistake to abandon the current bottom-up model—with investigator-initiated software development closely integrated with genomic research—in favor of a top-down model, dominated by large, centrally organized projects. Rather, a strategy is needed that embraces the strengths of our research-coupled model but promotes software quality and financial sustainability.

## Remedies: specific strategies

In keeping with the broad principles outlined above, I propose specific strategies in three major areas: grant funding, career development, and commercial development.

### Grant funding

There is clearly a need for continuing support for genomic software development from government grants, but the field would benefit substantially from improved grant opportunities, review criteria, and budget models. Some specific possibilities include:

- ▪ Changes to proposal formats and review criteria to focus attention on the engineering aspects of software projects that currently tend to be hidden in research proposals. For example, proposals with substantial software development components should be required to address in detail how software will be tested, distributed, and maintained, what user interfaces and documentation will be provided, how version control and bug-tracking will be managed, and how ongoing support will be offered to users. Explicit review criteria should be used to evaluate these features, and at least one suitably trained reviewer should examine each proposal with these criteria in mind.
- ▪ More government grant opportunities specifically focused on software development, with review criteria as described above. Review of these proposals should also allow for a reduced emphasis on novelty or innovation, as well as for the possibility that innovation might occur at the software design or implementation levels. A substantial fraction of these proposals should be awarded to individual investigator-initiated software projects, rather than being earmarked for large projects or consortia. Perhaps the best example of this type of funding in the US, at present, is the US National Science Foundation (NSF) Infrastructure Capacity for Biology program (formerly, Advances in Bioinformatics), but the funds devoted to this program are modest (Table 2), and they are spread across several types of infrastructure, including facilities, equipment, and biological collections.
- ▪ Many more government grant opportunities for the maintenance, support, or refinement of existing, widely used software tools. Programs of this kind were previously available from the NIH (for example, Continued Development and Maintenance of Software and Extended Development, Hardening & Dissemination of Technologies in Biomedical Computing, Informatics, & Big Data Science; Table 2) but have been discontinued. The new Investigator Initiated Research in Computational Genomics and Data Science program appears to be intended to replace them, in part, but it has a broader scope, and it is not clear how many awards will be funded through it. An important issue to address here is how to measure the impact and importance of existing software tools—through citations, downloads, expert opinion, or some other measure?
- ▪ Budget models that allow professional software developers to be paid competitive salaries from government grants. Current budgetary limits, such as the typical $250,000 per year in direct costs for a “modular” NIH grant, make it nearly impossible to pay these workers appropriately and still have funds for other necessities such as students, postdoctoral researchers, supplies, and portions of principal investigator salaries.
- ▪ Grant opportunities specifically designed to support computational scientists who wish to continue developing genomic software in a research setting, but who do not wish to serve as independent investigators. The US National Cancer Institute (NCI) Research Specialist (R50) award could serve as a model for such a program.
- ▪ More grant opportunities from private foundations and companies to support genomic software development. Private foundations, such as the W. M. Keck, Alfred P. Sloan, and Simons Foundations and the Wellcome Trust, have emerged as important auxiliary sources of scientific funding, but their support for projects in software development has so far been limited. Notable exceptions include the Data-Driven Discovery program from the Gordon & Betty Moore Foundation, the Collaborative Computational Tools for the Human Cell Atlas program from the Chan-Zuckerberg Initiative, and the Innovation in Cancer Informatics fund (Table 2).
- ▪ More grant opportunities to support community development for the kinds of distributed, open-source projects that have been so successful in computational genomics. For example, these grants could support workshops, “hackathons”, competitions, and challenges (such as CASP [158, 159] or DREAM [160]), creation of standardized benchmarks for testing, and public repositories for code and data.

### Career development

As noted above, a crucial barrier to genomic software development is the absence of stable and rewarding career paths for software developers working in academic research settings. Some institutions have been more effective than others at promoting the careers of these individuals—notable examples include the European Bioinformatics Institute, the Broad Institute, the UC Santa Cruz Genomics Institute, and the New York Genome Center—but improvements are needed broadly across the field. Aside from improved funding for salaries (above), the following ideas could be considered:

- ▪ Improved job descriptions, salary scales, and paths for career advancement, to allow recruitment and retention of first-rate software developers despite competition from industry. Software developers must be provided with clear paths from entry-level positions to jobs with increased pay, professional status, and/or leadership potential. In addition, academic job categories and descriptions should avoid blurring the distinctions among support roles; a software developer is not the same as a laboratory technician, a data analyst, or a systems administrator.
- ▪ Opportunities for continuing education. Software developers work in a fast-moving field, with new technologies continually emerging. They need to be able to attend their own conferences, workshops, and courses, just as researchers do. These activities would improve their productivity, generate and maintain excitement about their work, and help to create a sense of parity with workers on the research track.
- ▪ Institutional recognition of the accomplishments of software developers and other support staff. Some academic institutions bestow a seemingly limitless supply of awards and accolades on their faculty and students, but the critically important efforts of programmers, analysts, and technicians are too often overlooked. Recognizing these individuals is a natural way to help them feel valued.
- ▪ Encouragement for the development of forums for intellectual exchange among software developers and other staff members across an institution. For example, in-house seminars could be organized to focus on new programming languages, hardware resources, or other technologies, or to showcase the technical underpinnings of a new software release or data analysis.

### Commercial development

A third major area concerns the development of a sustainable commercial model to support aspects of software development that may be more efficiently, and more naturally, carried out in private companies than in academic research environments. Ideas to consider include:

- ▪ Grant mechanisms that make it easier to outsource software development, maintenance, and support to private companies, through contracts, consulting or service fees, or other arrangements, instead of implicitly encouraging academics to do this work for themselves (often poorly). For grant proposals that have a substantial software development component, investigators should perhaps be explicitly asked to present a rationale for their decision either to outsource the work or do it in-house. Institutions and granting agencies could facilitate outsourcing by providing lists of companies with various types of expertise.
- ▪ More proactive efforts by research institutions to spin off companies that develop genomic software. Many institutions have become much more active in encouraging start-ups in recent years, but development has been slow in the area of genomic software owing to uncertainty about business models. Nevertheless, if these efforts were paired with a push to outsource some grant-funded activities, perhaps the business models would begin to coalesce.
- ▪ More grants to support emerging genomic software companies, through mechanisms such as the Small Business Innovation Research (SBIR) program in the US (which does indeed fund some current software development activities).
- ▪ More efforts to expose graduate students and other trainees to commercial opportunities, including guidance on how to start their own companies, and benefit from institutional incubators and small business grants.

## Conclusions

Genomic software is now a fundamental component of the infrastructure for biological research. It is central to many thousands of research projects, costing many billions of dollars per year. Despite its crucial importance, genomic software development is generally funded at modest levels, primarily through a diffuse collection of government grants to individual researchers in academic research environments. This model is quite different from those adopted for other aspects of the infrastructure for life sciences research, such as public databases, which tend to be publicly funded but centrally organized, and laboratory equipment, which tends to be developed and marketed by private companies. The roots of these differences lie in the rapid growth of genomic software together with the emergence of the Internet, a generational change in the adoption of computers in biological research, and an affinity for the Open Source movement of the 1990s. Despite important strengths, the limitations of the current model are becoming increasingly apparent, with unreliable and hard-to-use software and inadequate maintenance and support, resulting in wasted time and money.

I have argued here that we need major changes in the way that we fund and carry out software development for genomics. In general, I propose measures intended to maintain the fundamental strengths of our current investigator-driven, research-coupled model of software development, but this model should be augmented with improved engineering practices, funding opportunities, career development, and commercial opportunities. These proposed measures would require action at multiple levels including in individual research groups, in institutions, and at funding agencies. They would clearly be costly. However, I believe that these costs are small in comparison to the many hidden costs of failing to offer a robust, reliable, efficient, and conveniently usable software infrastructure for genomics—costs that will only increase as the field grows in size and influence.

## References

[1] Wikipedia contributors. 40-foot telescope. Wikipedia, The Free Encyclopedia. 2019. https://en.wikipedia.org/wiki/40-foot_telescope Accessed 01/03/2019.

[2] https://www.yourgenome.org/. Accessed 01/03/2019.

[3] Wikipedia contributors. SPARCstation 1. Wikipedia, The Free Encyclopedia. 2019. Accessed 01/03/2019.

[4] Faulkner DV, Jurka J (1988). Multiple aligned sequence editor (MASE). Trends Biochem Sci.

[5] Wikipedia contributors. William Herschel. Wikipedia, The Free Encyclopedia. 2019. https://en.wikipedia.org/wiki/William_Herschel Accessed 01/03/2019.

[6] Morley K. Historical UK inflation calculator. 2019. http://inflation.iamkate.com/. Accessed 01/03/2019.

[7] Wikipedia contributors. Manhattan Project. Wikipedia, The Free Encyclopedia. 2019. https://en.wikipedia.org/wiki/Manhattan_Project Accessed 01/03/2019.

[8] US Inflation Calculator. Coinnews Media Group LLC. 2019. https://www.usinflationcalculator.com. Accessed 01/03/2019.

[9] Wikipedia contributors. Apollo program. In Wikipedia, The Free Encyclopedia. https://en.wikipedia.org/wiki/Apollo_program 2019. Accessed 01/03/2019.

[10] Wikipedia contributors. Space Shuttle program. Wikipedia, The Free Encyclopedia. 2019. https://en.wikipedia.org/wiki/Space_Shuttle_program Accessed 01/03/2019.

[11] Brumfiel G. LHC by the numbers. Nature. 2008. 10.1038/news.2008.1085.

[12] National Human Genome Research Institute .The Human Genome Project Completion: Frequently asked questions. 2019. https://www.genome.gov/human-genome-project/Completion-FAQ. Accessed 01/03/2019.

[13] Van Noorden R, Maher B, Nuzzo R (2014). The top 100 papers. Nature..

[14] Wren JD (2016). Bioinformatics programs are 31-fold over-represented among the highest impact scientific papers of the past two decades. Bioinformatics..

[15] Pearson WR, Lipman DJ (1988). Improved tools for biological sequence comparison. Proc Natl Acad Sci U S A.

[16] Chenna R, Sugawara H, Koike T, Lopez R, Gibson TJ, Higgins DG, Thompson JD (2003). Multiple sequence alignment with the Clustal series of programs. Nucleic Acids Res.

[17] Higgins DG, Sharp PM (1988). CLUSTAL: a package for performing multiple sequence alignment on a microcomputer. Gene..

[18] Jeanmougin F, Thompson JD, Gouy M, Higgins DG, Gibson TJ (1998). Multiple sequence alignment with Clustal X. Trends Biochem Sci.

[19] Larkin MA, Blackshields G, Brown NP, Chenna R, McGettigan PA, McWilliam H, Valentin F, Wallace IM, Wilm A, Lopez R (2007). Clustal W and Clustal X version 2.0. Bioinformatics..

[20] Thompson JD, Higgins DG, Gibson TJ (1994). CLUSTAL W: improving the sensitivity of progressive multiple sequence alignment through sequence weighting, position-specific gap penalties and weight matrix choice. Nucleic Acids Res.

[21] Altschul SF, Gish W, Miller W, Myers EW, Lipman DJ (1990). Basic local alignment search tool. J Mol Biol.

[22] Altschul SF, Madden TL, Schäffer AA, Zhang J, Zhang Z, Miller W, Lipman DJ (1997). Gapped BLAST and PSI-BLAST: a new generation of protein database search programs. Nucleic Acids Res.

[23] Eddy SR (1998). Profile hidden Markov models. Bioinformatics..

[24] Eddy SR (2011). Accelerated profile HMM searches. PLoS Comput Biol.

[25] Finn RD, Clements J, Eddy SR (2011). HMMER web server: interactive sequence similarity searching. Nucleic Acids Res.

[26] Notredame C, Higgins DG, Heringa J (2000). T-coffee: a novel method for fast and accurate multiple sequence alignment. J Mol Biol.

[27] Kent WJ (2002). BLAT--the BLAST-like alignment tool. Genome Res.

[28] Edgar RC (2004). MUSCLE: multiple sequence alignment with high accuracy and high throughput. Nucleic Acids Res.

[29] Katoh K, Asimenos G, Toh H (2009). Multiple alignment of DNA sequences with MAFFT. Methods Mol Biol.

[30] Katoh K, Kuma K, Toh H, Miyata T (2005). MAFFT version 5: improvement in accuracy of multiple sequence alignment. Nucleic Acids Res.

[31] Katoh K, Misawa K, Kuma K, Miyata T (2002). MAFFT: a novel method for rapid multiple sequence alignment based on fast Fourier transform. Nucleic Acids Res.

[32] Katoh K, Standley DM (2013). MAFFT multiple sequence alignment software version 7: improvements in performance and usability. Mol Biol Evol.

[33] Katoh K, Toh H (2008). Recent developments in the MAFFT multiple sequence alignment program. Brief Bioinform.

[34] Felsenstein J (1980). PHYLIP (Phylogeny Inference Package).

[35] Maddison WP, Maddison DR. MacClade, versions 3–4: Analysis of phylogeny and character evolution. 18. Swofford DL. PAUP*. Phylogenetic analysis using parsimony (and other methods). 1993. http://paup.phylosolutions.com/. Accessed 01/03/2019.

[36] Yang Z (1997). PAML: a program package for phylogenetic analysis by maximum likelihood. Comput Appl Biosci.

[37] Yang Z (2007). PAML 4: phylogenetic analysis by maximum likelihood. Mol Biol Evol.

[38] Kumar S, Stecher G, Tamura K (2016). MEGA7: molecular evolutionary genetics analysis version 7.0 for bigger datasets. Mol Biol Evol.

[39] Kumar S, Tamura K, Nei M (2004). MEGA3: integrated software for molecular evolutionary genetics analysis and sequence alignment. Brief Bioinform.

[40] Tamura K, Dudley J, Nei M, Kumar S (2007). MEGA4: molecular evolutionary genetics analysis (MEGA) software version 4.0. Mol Biol Evol.

[41] Tamura K, Peterson D, Peterson N, Stecher G, Nei M, Kumar S (2011). MEGA5: molecular evolutionary genetics analysis using maximum likelihood, evolutionary distance, and maximum parsimony methods. Mol Biol Evol.

[42] Tamura K, Stecher G, Peterson D, Filipski A, Kumar S (2013). MEGA6: molecular evolutionary genetics analysis version 6.0. Mol Biol Evol.

[43] Huelsenbeck JP, Ronquist F (2001). MRBAYES: Bayesian inference of phylogenetic trees. Bioinformatics..

[44] Ronquist F, Huelsenbeck JP (2003). MrBayes 3: Bayesian phylogenetic inference under mixed models. Bioinformatics..

[45] Ronquist F, Teslenko M, van der Mark P, Ayres DL, Darling A, Höhna S, Larget B, Liu L, Suchard MA, Huelsenbeck JP (2012). MrBayes 3.2: efficient Bayesian phylogenetic inference and model choice across a large model space. Syst Biol.

[46] Maddison DR, Maddison WP. Mesquite: a modular system for evolutionary analysis. 2003. http://mesquiteproject.org. Accessed 01/03/2019.

[47] Guindon S, Delsuc F, Dufayard JF, Gascuel O (2009). Estimating maximum likelihood phylogenies withPhyML. Methods Mol Biol.

[48] Guindon S, Dufayard JF, Lefort V, Anisimova M, Hordijk W, Gascuel O (2010). New algorithms and methods to estimate maximum-likelihood phylogenies: assessing the performance of PhyML 3.0. Syst Biol.

[49] Guindon S, Gascuel O (2003). A simple, fast, and accurate algorithm to estimate large phylogenies by maximum likelihood. Syst Biol.

[50] Guindon S, Lethiec F, Duroux P, Gascuel O (2005). PHYML online--a web server for fast maximum likelihood-based phylogenetic inference. Nucleic Acids Res.

[51] Hubisz MJ, Pollard KS, Siepel A (2011). PHAST and RPHAST: phylogenetic analysis with space/time models. Brief Bioinform.

[52] Pollard KS, Hubisz MJ, Rosenbloom KR, Siepel A (2010). Detection of nonneutral substitution rates on mammalian phylogenies. Genome Res.

[53] Siepel A, Bejerano G, Pedersen JS, Hinrichs AS, Hou M, Rosenbloom K, Clawson H, Spieth J, Hillier LW, Richards S (2005). Evolutionarily conserved elements in vertebrate, insect, worm, and yeast genomes. Genome Res.

[54] Siepel A, Haussler D (2004). Combining phylogenetic and hidden Markov models in biosequence analysis. J Comput Biol.

[55] Siepel A, Haussler D (2004). Phylogenetic estimation of context-dependent substitution rates by maximum likelihood. Mol Biol Evol.

[56] Stamatakis A (2006). RAxML-VI-HPC: maximum likelihood-based phylogenetic analyses with thousands of taxa and mixed models. Bioinformatics..

[57] Stamatakis A (2014). RAxML version 8: a tool for phylogenetic analysis and post-analysis of large phylogenies. Bioinformatics..

[58] Stamatakis A, Hoover P, Rougemont J (2008). A rapid bootstrap algorithm for the RAxML web servers. Syst Biol.

[59] Stamatakis A, Ludwig T, Meier H (2005). RAxML-III: a fast program for maximum likelihood-based inference of large phylogenetic trees. Bioinformatics..

[60] Pond SLK, Muse SV, Nielsen R (2005). HyPhy: hypothesis testing using phylogenies. Statistical methods in molecular evolution.

[61] Bouckaert R, Heled J, Kühnert D, Vaughan T, Wu CH, Xie D, Suchard MA, Rambaut A, Drummond AJ (2014). BEAST 2: a software platform for Bayesian evolutionary analysis. PLoS Comput Biol.

[62] Drummond AJ, Rambaut A (2007). BEAST: Bayesian evolutionary analysis by sampling trees. BMC Evol Biol.

[63] Price MN, Dehal PS, Arkin AP (2009). FastTree: computing large minimum evolution trees with profiles instead of a distance matrix. Mol Biol Evol.

[64] Price MN, Dehal PS, Arkin AP (2010). FastTree 2--approximately maximum-likelihood trees for large alignments. PLoS One.

[65] Bailey TL, Boden M, Buske FA, Frith M, Grant CE, Clementi L, Ren J, Li WW, Noble WS (2009). MEME SUITE: tools for motif discovery and searching. Nucleic Acids Res.

[66] Bailey TL, Elkan C (1994). Fitting a mixture model by expectation maximization to discover motifs in biopolymers. Proc Int Conf Intell Syst Mol Biol.

[67] Bailey TL, Elkan C (1995). Unsupervised learning of multiple motifs in biopolymers using expectation maximization. Mach Learn.

[68] Bailey TL, Williams N, Misleh C, Li WW (2006). MEME: discovering and analyzing DNA and protein sequence motifs. Nucleic Acids Res.

[69] Grant CE, Bailey TL, Noble WS (2011). FIMO: scanning for occurrences of a given motif. Bioinformatics..

[70] Burge C, Karlin S (1997). Prediction of complete gene structures in human genomic DNA. J Mol Biol.

[71] Lowe TM, Eddy SR (1997). tRNAscan-SE: a program for improved detection of transfer RNA genes in genomic sequence. Nucleic Acids Res.

[72] Gruber AR, Lorenz R, Bernhart SH, Neuböck R, Hofacker IL (2008). The Vienna RNA websuite. Nucleic Acids Res.

[73] Hofacker IL (2003). Vienna RNA secondary structure server. Nucleic Acids Res.

[74] Lorenz R, Bernhart SH, Höner Zu Siederdissen C, Tafer H, Flamm C, Stadler PF, Hofacker IL (2011). ViennaRNA Package 2.0. Algorithms Mol Biol.

[75] Clamp M, Cuff J, Searle SM, Barton GJ (2004). The Jalview Java alignment editor. Bioinformatics..

[76] Waterhouse AM, Procter JB, Martin DM, Clamp M, Barton GJ (2009). Jalview version 2--a multiple sequence alignment editor and analysis workbench. Bioinformatics..

[77] Eisen MB, Spellman PT, Brown PO, Botstein D (1998). Cluster analysis and display of genome-wide expression patterns. Proc Natl Acad Sci U S A.

[78] Fujita PA, Rhead B, Zweig AS, Hinrichs AS, Karolchik D, Cline MS, Goldman M, Barber GP, Clawson H, Coelho A (2011). The UCSC genome browser database: update 2011. Nucleic Acids Res.

[79] Karolchik D, Beartsch R, Diekhans M, Furey TS, Hinrichs A, Lu YT, Roskin KM, Schwartz M, Sugnet CW, Thomas DJ (2003). The UCSC genome browser database. Nucleic Acids Res.

[80] Karolchik D, Hinrichs AS, Furey TS, Roskin KM, Sugnet CW, Haussler D, Kent WJ (2004). The UCSC table browser data retrieval tool. Nucleic Acids Res.

[81] Kent WJ, Sugnet CW, Furey TS, Roskin KM, Pringle TH, Zahler AM, Haussler D (2002). The human genome browser at UCSC. Genome Res.

[82] Meyer LR, Zweig AS, Hinrichs AS, Karolchik D, Kuhn RM, Wong M, Sloan CA, Rosenbloom KR, Roe G, Rhead B (2013). The UCSC genome browser database: extensions and updates 2013. Nucleic Acids Res.

[83] Cunningham F, Amode MR, Barrell D, Beal K, Billis K, Brent S, Carvalho-Silva D, Clapham P, Coates G, Fitzgerald S (2015). Ensembl 2015. Nucleic Acids Res.

[84] Flicek P, Ahmed I, Amode MR, Barrell D, Beal K, Brent S, Carvalho-Silva D, Clapham P, Coates G, Fairley S (2013). Ensembl 2013. Nucleic Acids Res.

[85] Flicek P, Amode MR, Barrell D, Beal K, Billis K, Brent S, Carvalho-Silva D, Clapham P, Coates G, Fitzgerald S (2014). Ensembl 2014. Nucleic Acids Res.

[86] Flicek P, Amode MR, Barrell D, Beal K, Brent S, Carvalho-Silva D, Clapham P, Coates G, Fairley S, Fitzgerald S (2012). Ensembl 2012. Nucleic Acids Res.

[87] Yates A, Akanni W, Amode MR, Barrell D, Billis K, Carvalho-Silva D, Cummins C, Clapham P, Fitzgerald S, Gil L (2016). Ensembl 2016. Nucleic Acids Res.

[88] Cline MS, Smoot M, Cerami E, Kuchinsky A, Landys N, Workman C, Christmas R, Avila-Campilo I, Creech M, Gross B (2007). Integration of biological networks and gene expression data using Cytoscape. Nat Protoc.

[89] Shannon P, Markiel A, Ozier O, Baliga NS, Wang JT, Ramage D, Amin N, Schwikowski B, Ideker T (2003). Cytoscape: a software environment for integrated models of biomolecular interaction networks. Genome Res.

[90] Smoot ME, Ono K, Ruscheinski J, Wang PL, Ideker T (2011). Cytoscape 2.8: new features for data integration and network visualization. Bioinformatics..

[91] Robinson JT, Thorvaldsdóttir H, Winckler W, Guttman M, Lander ES, Getz G, Mesirov JP (2011). Integrative genomics viewer. Nat Biotechnol.

[92] Falush D, Stephens M, Pritchard JK (2003). Inference of population structure using multilocus genotype data: linked loci and correlated allele frequencies. Genetics.

[93] Pritchard JK, Stephens M, Donnelly P (2000). Inference of population structure using multilocus genotype data. Genetics.

[94] Scheet P, Stephens M (2006). A fast and flexible statistical model for large-scale population genotype data: applications to inferring missing genotypes and haplotypic phase. Am J Hum Genet.

[95] Stephens M, Scheet P (2005). Accounting for decay of linkage disequilibrium in haplotype inference and missing-data imputation. Am J Hum Genet.

[96] Stephens M, Smith NJ, Donnelly P (2001). A new statistical method for haplotype reconstruction from population data. Am J Hum Genet.

[97] Hudson RR (2002). Generating samples under a Wright-fisher neutral model of genetic variation. Bioinformatics..

[98] Adzhubei Ivan, Jordan Daniel M., Sunyaev Shamil R. (2013). Predicting Functional Effect of Human Missense Mutations Using PolyPhen-2. Current Protocols in Human Genetics.

[99] Adzhubei IA, Schmidt S, Peshkin L, Ramensky VE, Gerasimova A, Bork P, Kondrashov AS, Sunyaev SR (2010). A method and server for predicting damaging missense mutations. Nat Methods.

[100] Ramensky V, Bork P, Sunyaev S (2002). Human non-synonymous SNPs: server and survey. Nucleic Acids Res.

[101] Kumar P, Henikoff S, Ng PC (2009). Predicting the effects of coding non-synonymous variants on protein function using the SIFT algorithm. Nat Protoc.

[102] Ng PC, Henikoff S (2003). SIFT: predicting amino acid changes that affect protein function. Nucleic Acids Res.

[103] Price AL, Patterson NJ, Plenge RM, Weinblatt ME, Shadick NA, Reich D (2006). Principal components analysis corrects for stratification in genome-wide association studies. Nat Genet.

[104] Chang CC, Chow CC, Tellier LC, Vattikuti S, Purcell SM, Lee JJ (2015). Second-generation PLINK: rising to the challenge of larger and richer datasets. Gigascience..

[105] Purcell S, Neale B, Todd-Brown K, Thomas L, Ferreira MA, Bender D, Maller J, Sklar P, de Bakker PI, Daly MJ (2007). PLINK: a tool set for whole-genome association and population-based linkage analyses. Am J Hum Genet.

[106] Bradbury PJ, Zhang Z, Kroon DE, Casstevens TM, Ramdoss Y, Buckler ES (2007). TASSEL: software for association mapping of complex traits in diverse samples. Bioinformatics..

[107] Browning BL, Browning SR (2009). A unified approach to genotype imputation and haplotype-phase inference for large data sets of trios and unrelated individuals. Am J Hum Genet.

[108] Browning SR, Browning BL (2007). Rapid and accurate haplotype phasing and missing-data inference for whole-genome association studies by use of localized haplotype clustering. Am J Hum Genet.

[109] Howie BN, Donnelly P, Marchini J (2009). A flexible and accurate genotype imputation method for the next generation of genome-wide association studies. PLoS Genet.

[110] Marchini J, Howie B, Myers S, McVean G, Donnelly P (2007). A new multipoint method for genome-wide association studies by imputation of genotypes. Nat Genet.

[111] Danecek P, Auton A, Abecasis G, Albers CA, Banks E, DePristo MA, Handsaker RE, Lunter G, Marth GT, Sherry ST (2011). The variant call format and VCFtools. Bioinformatics..

[112] Kircher M, Witten DM, Jain P, O’Roak BJ, Cooper GM, Shendure J (2014). A general framework for estimating the relative pathogenicity of human genetic variants. Nat Genet.

[113] Ashburner M, Ball CA, Blake JA, Botstein D, Butler H, Cherry JM, Davis AP, Dolinski K, Dwight SS, Eppig JT (2000). Gene ontology: tool for the unification of biology. Gene Ontol Consortium Nat Genet.

[114] Subramanian A, Tamayo P, Mootha VK, Mukherjee S, Ebert BL, Gillette MA, Paulovich A, Pomeroy SL, Golub TR, Lander ES (2005). Gene set enrichment analysis: a knowledge-based approach for interpreting genome-wide expression profiles. Proc Natl Acad Sci U S A.

[115] Feng J, Liu T, Qin B, Zhang Y, Liu XS (2012). Identifying ChIP-seq enrichment using MACS. Nat Protoc.

[116] Zhang Y, Liu T, Meyer CA, Eeckhoute J, Johnson DS, Bernstein BE, Nusbaum C, Myers RM, Brown M, Li W (2008). Model-based analysis of ChIP-Seq (MACS). Genome Biol.

[117] Kim D, Pertea G, Trapnell C, Pimentel H, Kelley R, Salzberg SL (2013). TopHat2: accurate alignment of transcriptomes in the presence of insertions, deletions and gene fusions. Genome Biol.

[118] Trapnell C, Pachter L, Salzberg SL (2009). TopHat: discovering splice junctions with RNA-Seq. Bioinformatics..

[119] Trapnell C, Roberts A, Goff L, Pertea G, Kim D, Kelley DR, Pimentel H, Salzberg SL, Rinn JL, Pachter L (2012). Differential gene and transcript expression analysis of RNA-seq experiments with TopHat and cufflinks. Nat Protoc.

[120] Trapnell C, Williams BA, Pertea G, Mortazavi A, Kwan G, van Baren MJ, Salzberg SL, Wold BJ, Pachter L (2010). Transcript assembly and quantification by RNA-Seq reveals unannotated transcripts and isoform switching during cell differentiation. Nat Biotechnol.

[121] Ernst J, Kellis M (2010). Discovery and characterization of chromatin states for systematic annotation of the human genome. Nat Biotechnol.

[122] Ernst J, Kellis M (2012). ChromHMM: automating chromatin-state discovery and characterization. Nat Methods.

[123] Ernst J, Kheradpour P, Mikkelsen TS, Shoresh N, Ward LD, Epstein CB, Zhang X, Wang L, Issner R, Coyne M (2011). Mapping and analysis of chromatin state dynamics in nine human cell types. Nature.

[124] Quinlan AR (2014). BEDTools: the Swiss-army tool for genome feature analysis. Curr Protoc Bioinformatics.

[125] Quinlan AR, Hall IM (2010). BEDTools: a flexible suite of utilities for comparing genomic features. Bioinformatics..

[126] Robinson MD, McCarthy DJ, Smyth GK (2010). edgeR: a Bioconductor package for differential expression analysis of digital gene expression data. Bioinformatics.

[127] Grabherr MG, Haas BJ, Yassour M, Levin JZ, Thompson DA, Amit I, Adiconis X, Fan L, Raychowdhury R, Zeng Q (2011). Full-length transcriptome assembly from RNA-Seq data without a reference genome. Nat Biotechnol.

[128] Anders S, Huber W. Differential expression of RNA-Seq data at the gene level-the DESeq package. 2012. https://bioconductor.org/packages/release/bioc/vignettes/DESeq/inst/doc/DESeq.pdf. Accessed 01/03/2019.

[129] Love MI, Huber W, Anders S (2014). Moderated estimation of fold change and dispersion for RNA-seq data with DESeq2. Genome Biol.

[130] Staden R (1996). The Staden sequence analysis package. Mol Biotechnol.

[131] Staden R, Beal KF, Bonfield JK (2000). The Staden package, 1998. Methods Mol Biol.

[132] Bonfield JK, Smith K, Staden R (1995). A new DNA sequence assembly program. Nucleic Acids Res.

[133] Staden R (1982). An interactive graphics program for comparing and aligning nucleic acid and amino acid sequences. Nucleic Acids Res.

[134] Staden R (1984). Computer methods to locate signals in nucleic acid sequences. Nucleic Acids Res.

[135] Ewing B, Hillier L, Wendl MC, Green P (1998). Base-calling of automated sequencer traces using phred. I Accuracy assessment. Genome Res.

[136] Ewing B, Green P (1998). Base-calling of automated sequencer traces using phred. II Error probabilities. Genome Res.

[137] Li H, Ruan J, Durbin R (2008). Mapping short DNA sequencing reads and calling variants using mapping quality scores. Genome Res.

[138] Butler J, MacCallum I, Kleber M, Shlyakhter IA, Belmonte MK, Lander ES, Nusbaum C, Jaffe DB (2008). ALLPATHS: de novo assembly of whole-genome shotgun microreads. Genome Res.

[139] Gnerre S, MacCallum I, Przybylski D, Ribeiro FJ, Burton JN, Walker BJ, Sharpe T, Hall G, Shea TP, Sykes S (2011). High-quality draft assemblies of mammalian genomes from massively parallel sequence data. Proc Natl Acad Sci U S A.

[140] Zerbino DR, Birney E (2008). Velvet: algorithms for de novo short read assembly using de Bruijn graphs. Genome Res.

[141] Langmead B, Salzberg SL (2012). Fast gapped-read alignment with bowtie 2. Nat Methods.

[142] Langmead B, Trapnell C, Pop M, Salzberg SL (2009). Ultrafast and memory-efficient alignment of short DNA sequences to the human genome. Genome Biol.

[143] Li H, Durbin R (2009). Fast and accurate short read alignment with burrows-wheeler transform. Bioinformatics..

[144] Li R, Yu C, Li Y, Lam TW, Yiu SM, Kristiansen K, Wang J (2009). SOAP2: an improved ultrafast tool for short read alignment. Bioinformatics..

[145] Li H, Handsaker B, Wysoker A, Fennell T, Ruan J, Homer N, Marth G, Abecasis G, Durbin R (2009). 1000 genome project data processing subgroup. The sequence alignment/map format and SAMtools. Bioinformatics..

[146] Simpson JT, Wong K, Jackman SD, Schein JE, Jones SJ, Birol I (2009). ABySS: a parallel assembler for short read sequence data. Genome Res.

[147] McKenna A, Hanna M, Banks E, Sivachenko A, Cibulskis K, Kernytsky A, Garimella K, Altshuler D, Gabriel S, Daly M (2010). The genome analysis toolkit: a MapReduce framework for analyzing next-generation DNA sequencing data. Genome Res.

[148] Li R, Zhu H, Ruan J, Qian W, Fang X, Shi Z, Li Y, Li S, Shan G, Kristiansen K (2010). De novo assembly of human genomes with massively parallel short read sequencing. Genome Res.

[149] Luo R, Liu B, Xie Y, Li Z, Huang W, Yuan J, He G, Chen Y, Pan Q, Liu Y (2012). SOAPdenovo2: an empirically improved memory-efficient short-read de novo assembler. Gigascience..

[150] Dobin A, Davis CA, Schlesinger F, Drenkow J, Zaleski C, Jha S, Batut P, Chaisson M, Gingeras TR (2013). STAR: ultrafast universal RNA-seq aligner. Bioinformatics..

[151] Dobin A, Gingeras TR (2015). Mapping RNA-seq reads with STAR. Curr Protoc Bioinformatics.

[152] https://aminer.org/open-academic-graph. Accessed 01/03/2019.

[153] National Center for Science and Engineering Statistics. Federal R&D obligations increase 3% in FY 2017: Research obligations decrease slightly while those for development increase 7%. InfoBriefs. 2018;NSF 18–311.

[154] Wikipedia contributors. List of countries by research and development spending. Wikipedia, The Free Encyclopedia. 2019. https://en.wikipedia.org/wiki/List_of_countries_by_research_and_development_spending Accessed 01/03/2019.

[155] https://www.payscale.com/research/US/Location=San-Francisco-CA/Salary. Accessed 01/03/2019.

[156] Mangul S*,* Mosqueiro T, Abdil RJ, Duong D, Mitchell K, Sarwal V, Hill B, Brito J, Littman RJ, Statz B, et al. A comprehensive analysis of the usability and archival stability of omics computational tools and resources. bioRxiv. 2018. doi: 10.1101/452532 10.1371/journal.pbio.3000333 PMC6605654 31220077

[157] Raymond E (1999). The cathedral and the bazaar. Knowledge Technology Policy.

[158] Moult J (2005). A decade of CASP: progress, bottlenecks and prognosis in protein structure prediction. Curr Opin Struct Biol.

[159] CASP. http://predictioncenter.org/. Accessed 1 Mar 2019.

[160] DREAM Challenge. http://dreamchallenges.org/. Accessed 1 Mar 2019.

